# The Value of Neutrophil-To-Lymphocyte Ratio for Evaluating Blood Stream Infection Caused by Carbapenem-Resistant *Klebsiella pneumoniae*: A Retrospective Cohort Study

**DOI:** 10.3389/fmed.2022.832655

**Published:** 2022-03-07

**Authors:** Heng Wu, Yihan Mao, Xiaoxing Du, Feng Zhao, Yan Jiang, Yunsong Yu

**Affiliations:** ^1^Department of Infectious Diseases, Sir Run Run Shaw Hospital, Zhejiang University School of Medicine, Hangzhou, China; ^2^Key Laboratory of Microbial Technology and Bioinformatics of Zhejiang Province, Hangzhou, China; ^3^Regional Medical Center for National Institute of Respiratory Diseases, Sir Run Run Shaw Hospital, Zhejiang University School of Medicine, Hangzhou, China; ^4^Department of General Practice, Sir Run Run Shaw Hospital, Zhejiang University School of Medicine, Hangzhou, China; ^5^Department of Clinical Laboratory, Sir Run Run Shaw Hospital, Zhejiang University School of Medicine, Hangzhou, China

**Keywords:** neutrophil-to-lymphocyte ratio, carbapenem-resistant *Klebsiella pneumoniae*, blood stream infection, prognosis, therapy strategies

## Abstract

**Background:**

The neutrophil-to-lymphocyte ratio (NLR) is a useful marker of inflammation. However, the prognostic function of the NLR in patients with carbapenem-resistant *Klebsiella pneumoniae* (CRKP) blood stream infection (BSI) remains largely unknown. The aim of this study was to explore the potential relationship between the NLR and mortality in these patients.

**Methods:**

We performed a retrospective cohort study based on data retrieved from the computerized patient record system in a tertiary hospital from 1 January 2017 to 31 October, 2020. A total of 134 inpatients with CRKP BSI were enrolled in this study, including 54 fatal cases and 80 survival cases, 28 days after the onset of CRKP BSI. A logistic analysis was performed to assess the association between the NLR on the 4th day and 28-day mortality. Multivariate analyses were used to control for the confounders.

**Results:**

The overall 28-day mortality rate of patients with a CRKP BSI episode was 40.3% (54/134). We conducted a multivariate analysis of the data of 134 patients and found that the NLR on the 4th day [odds ratio (OR) 1.148, 95% confidence interval (CI) 1.076–1.225, *p* < 0.001] and antibiotic exposure before BSI onset (OR 3.847, 95% CI 1.322–11.196, *p* = 0.013) were independent risk factors for 28-day mortality of patients with CRKP BSI, while appropriate initial therapy (AIT, OR 0.073, 95% CI 0.017–0.307, *p* < 0.001) was an independent protective factor. Among patients treated with AITs, the Cox proportional hazards regression analysis revealed a significant difference in prognosis (p = 0.006) between the ceftazidime/avibactam contained (CAZ) group and non CAZ-AVI groups. After dividing the non CAZ-AVI group into the tigecycline (TGC), colistin (COL), and TGC + COL groups, there were no differences between the CAZ-AVI group and the TGC group (*p* = 0.093), but CAZ-AVI group showed lower 28-day mortality than COL (*p* = 0.002) and TGC + COL (*p* = 0.002) groups. Meanwhile, there was no difference in NLR on the 1st day (*p* = 0.958) of patients in different groups but significant difference in NLR on the 4th day (*p* = 0.047).

**Conclusions:**

The NLR on the 4th day is a readily available and independent prognostic biomarker for patients with CRKP BSI. This marker may have the potential for use in evaluating the efficacy of different anti-infection therapy strategies at an early stage.

## Introduction

*Klebsiella pneumoniae* is one of the most common bacteria in the class Enterobacteriales; it is ubiquitous and can cause nosocomial infections, such as pneumonia, urinary tract infection, catheter-related infection, and blood stream infection (BSI) (1, 2). *K. pneumoniae* isolates can develop resistance by producing extended-spectrum β-lactamases (ESBLs) (3, 4). Carbapenems are the first-line therapy for severe infections caused by ESBL-producing KPs (5). However, with the increasing clinical use of carbapenems over the last few years, carbapenem-resistant *K. pneumoniae* (CRKP) has risen at an alarming rate, and is considered a serious threat to human health worldwide. It has been recorded in the China antimicrobial surveillance network that from 2005 to 2021, the proportion of *K. pneumoniae* isolates resistant to imipenem increased from 3.0 to 25.5% in China (http://www.chinets.com/).

Patients infected with CRKP have higher mortality rates than those infected with carbapenem-susceptible *Klebsiella pneumonia* (CSKP) (6). It was reported that the mortality of patients with CRKP infection, mainly BSI, was up to 70% (7), and a high readmission rate of survivors (~72%) within 90 days of discharge was reported (8). The proposed hypotheses for this increased mortality include (1) severe comorbidities, (2) increased virulence of carbapenemase-producing strains, (3) low effectiveness and high toxicity of drugs used for treatment of these infections, and (4) a low probability of receiving appropriate initial antibiotic therapy (9).

Since CRKP BSIs would result in worse clinical outcomes, early and accurate evaluation is essential for the treatment and prognosis of these patients. The neutrophil-to-lymphocyte ratio (NLR) is a measure of systemic inflammation derived from the white blood cell (WBC) count, one of the most common infection markers. It has been used as a predictor of cardiovascular diseases (10) and cancer (11). Zahorec proposed the use of the NLR as an additional infection marker in clinical intensive care unit practice based on the phenomenon that the physiological immune response of circulating leukocytes to various stressful events is often characterized by an increase in neutrophil counts and a decline in lymphocyte counts (12). Additionally, according to Acute Physiology and Chronic Health Evaluation II (APACHE II) and Sepsis-related Organ Failure Assessment (SOFA) scores, it was found that NLR correlated well with the severity of disease and outcome (13). de Jager et al. evaluated the performance of NLR and other markers of infection in predicting bacteraemia in adults and found it to be a better predictor than C-reaction protein (CRP) levels and WBC counts (14).

Nevertheless, the value of the NLR in predicting the prognosis of patients with CRKP BSI is rarely reported. Thus, we performed a cohort study to evaluate NLR as a predictor of the prognosis of these patients.

## Methods and Material

### Ethics

All isolates present in this study were stored in the Department of Microbiology of a tertiary hospital, Sir Run Run Shaw Hospital, Zhejiang University School of Medicine. The study was approved by the ethical research committee of Sir Run Run Shaw Hospital, College of Medicine, Zhejiang University. The ethics committee approved the waiver of patients' informed consent, with the justification that this was a retrospective and analytical study whose information was obtained from medical records and that the data were stripped of identifying information and anonymously analyzed. The study was performed in accordance with the Declaration of Helsinki and its amendments.

Privacy statement: the authors guarantee confidentiality of the patient data (Ethics No. 20170301-3).

### Study Design and Data Source

**Figure 1:**
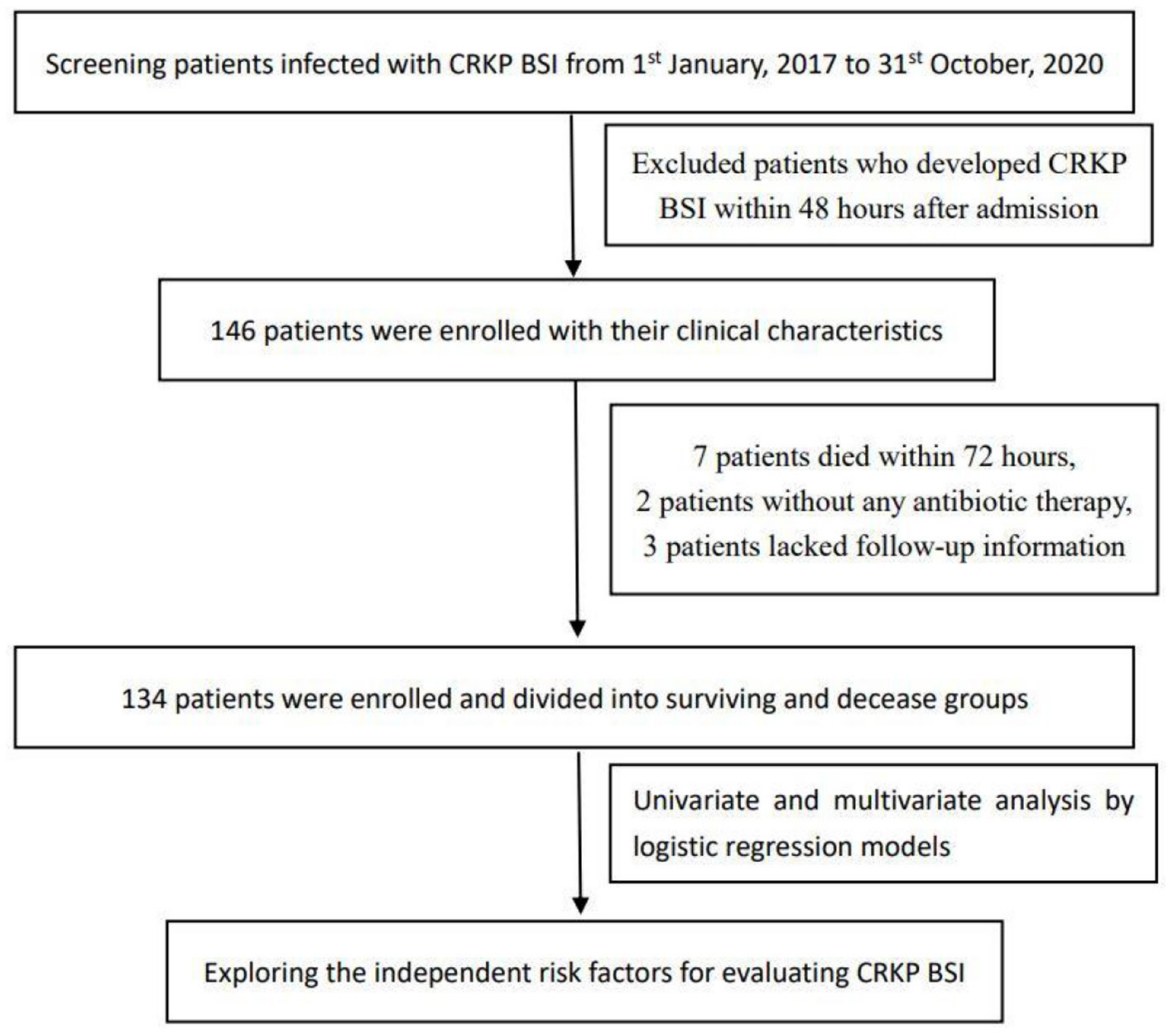
Flowchart of the study design.

We performed a single-center, retrospective cohort study based on data retrieved from the Computerized Patient Record System of the Sir Run Run Shaw Hospital, Zhejiang University School of Medicine, Hangzhou, China. Patients aged ≥18 years who were confirmed to present with CRKP BSI were included from January 1, 2017 to October 31, 2020. Episodes of CRKP BSI were identified based on blood culture results. All patients with CRKP BSI were followed up for 28 days and were divided into surviving and deceased groups. The database contains comprehensive clinical data, including patient characteristics, laboratory outcomes, clinical diagnoses, and medical records (Figure 1).

### Inclusion and Exclusion Criteria

We included patients with CRKP BSI from the database with their medical records. The exclusion criteria were as follows: (I) non-adult patients, (II) patients who developed CRKP BSI within 48 hours after admission, (III) patients who died within 72 h after the first positive blood culture or those with no antibiotic therapy records, and (IV) lack of records regarding laboratory examinations on the onset of BSI or the 4th (±1) day. A total of 134 CRKP BSI inpatients were included in this study, including 54 fatal cases and 80 survival cases on day 28 after the onset of CRKP BSI.

### Definitions

CRKP BSI was defined as a positive blood culture for *K. pneumoniae* with resistance to any carbapenem combined with symptoms of infection and elevated CRP or procalcitonin (PCT), including those sampled from a peripherally inserted central catheter or central venous catheter. The date when the first positive blood culture was sampled was considered to be the onset of BSI and recorded as the first day. Renal damage was defined as a creatinine clearance of <30 mL/min, while liver damage was defined as an alanine transaminase or aspartate aminotransferase level >3-fold of the upper normal limit. Antibiotic exposure meant that antibiotics were administered intravenously or orally for more than 48 h within the past 14 days. Appropriate initial therapy (AIT) was defined as antibiotics, confirmed to be active against CRKP by a susceptibility test, administered within 72 hours after BSI onset. All-cause mortality was defined as death from any cause during hospitalization.

### Statistical Analysis

Continuous variables are presented as mean ± standard deviation or median (interquartile range), and categorical variables are presented as frequencies and percentages. Continuous variables with an abnormal distribution were compared using Student's *t*-test or Mann–Whitney *U*-test. Continuous variables in more than two groups were compared using Analysis of Variance (ANOVA). The chi-square or Fisher's exact tests were used to compare categorical variables. Risk factors for mortality from CRKP BSI were analyzed using binary logistic regression. Variables with *p* < 0.10 and clinical relevance in the univariate analysis were selected for logistic regression models for the multivariate analysis to evaluate risk factors for 28-day mortality of CRKP BSI.

## Results

### Clinical and Demographic Characteristic of Patients With CRKP BSI and Risk Factors for 28-Day Mortality

The overall 28-day mortality rate of patients with a CRKP BSI episode was 40.3% (54/134). The clinical and demographic characteristics of cohort patients with CRKP BSI isolates are shown in Table 1 according to the 28-day survival status.

**Table 1:** Clinical and demographic characteristics of 134 patients with CRKP BSI and risk factors for 28-day mortality.

	**Total *N* = 134**	**28d-Surviving *N* = 80**	**28d-Deceased *N* = 54**	***p*-value**
**Demographic variables**				
Age	66 (55, 73)	66 (52, 73)	68 (59, 73)	0.577
Male	92 (68.6)	58 (72.5)	34 (63.0)	0.243
Body mass index (<18.5 or >24)	68 (50.7)	41 (51.2)	27 (50.0)	0.887
**Underlying diseases**				
Hypertension	56 (41.8)	36 (45.0)	20 (37.3)	0.359
Diabetes mellitus	27 (20.1)	18 (22.5)	9 (25.9)	0.208
Cardiovascular and cerebrovascular diseases	15 (11.2)	9 (11.2)	6 (18.5)	0.237
Chronic lung diseases	32 (23.9)	20 (25.0)	12 (35.2)	0.203
Liver failure	15 (11.2)	10 (12.5)	5 (14.8)	0.700
Renal failure	11 (8.2)	6 (7.5)	5 (14.8)	0.175
Solid tumor	34 (25.4)	25 (31.2)	9 (25.9)	0.506
Hematological malignancy	6 (4.5)	3 (3.8)	3 (9.3)	0.267
Immunosuppressive therapy	11 (8.2)	6 (7.5)	5 (14.8)	0.175
Age-adjusted Charlson comorbidity index	4.90 ± 2.45	4.55 ± 2.34	5.41 ± 2.54	**0.047**
**Two weeks before the onset of BSI**				
ICU stay	72 (53.0)	40 (50.0)	32 (59.2)	0.292
Antibiotic exposure	56 (41.8)	24 (30.0)	32 (59.2)	**0.001**
Tigecycline	3 (5.4)	1 (4.2)	2 (6.2)	0.999
Quinolones	1 (1.8)	1 (4.2)	0 (0)	0.429
β-Lactam/lactamase combinations	23 (41.1)	11 (45.8)	12 (37.5)	0.530
Carbapenems	29 (33.9)	11 (45.8)	18 (56.2)	0.440
**The day of BSI onset**				
APACHE II	20 (16, 26)	17 (12, 23)	22 (19, 29)	**<0.001**
SOFA	6 (4, 9)	5 (3, 8)	8 (5, 10)	**<0.001**
Hospitalized days before BSI onset	14 (2, 23)	12 (1, 20)	18 (11, 35)	**0.041**
Appropriate Initial Therapy	112 (83.6)	75 (93.8)	37 (68.5)	**0.001**
White blood cell (10^9^/L)	13.36 ± 7.77	13.62 ± 7.90	12.97 ± 7.64	0.637
Neutrophil to lymphocyte count ratio	30.97 ± 38.16	28.69 ± 34.14	34.35 ± 43.56	0.402
C-reaction protein (mg/L)	133.64 ± 78.95	134.12 ± 82.30	132.93 ± 74.47	0.932
Procalcitonin (ng/mL)	19.24 ± 40.21	20.12 ± 42.72	17.94 ± 36.57	0.769
Hemoglobin (g/L)	85.04 ± 22.06	86.16 ± 22.18	83.39 ± 22.00	0.477
Platelet (10^9^/L)	154.12 ± 117.31	163.29 ± 116.10	140.54 ± 118.87	0.272
Hematocrit	26.11 ± 7.58	26.07 ± 6.72	26.17 ± 8.76	0.939
Alanine transaminase (U/L)	58.63 ± 128.10	56.20 ± 124.28	62.24 ± 134.67	0.790
Aspartate aminotransferase (U/L)	81.49 ± 222.38	90.38 ± 272.81	68.48 ± 115.83	0.579
Total bilirubin (μmol/L)	61.45 ± 79.84	54.00 ± 75.97	72.49 ± 84.76	0.190
Direct bilirubin (μmol/L)	44.52 ± 61.16	38.46 ± 57.70	53.49 ± 65.48	0.175
Albumin (g/L)	29.10 ± 4.70	29.73 ± 4.79	28.17 ± 4.43	0.059
Creatinine (μmol/L)	135.40 ± 147.77	132.40 ± 150.45	139.83 ± 144.99	0.776
**The 4th day after BSI onset**				
APACHE II	18 (13, 24)	15 (9, 20)	23 (19, 29)	**<0.001**
SOFA	5 (4, 8)	4 (2, 7)	8 (5, 10)	**<0.001**
White blood cell count (10^9^/L)	10.56 ± 6.29	9.36 ± 4.98	12.34 ± 7.54	**0.007**
Neutrophil-to-lymphocyte ratio	19.06 ± 24.72	9.42 ± 6.41	33.34 ± 33.53	**<0.001**
C-reaction protein (mg/L)	109.00 ± 69.16	87.08 ± 62.65	141.48 ± 65.97	**<0.001**
Procalcitonin (ng/mL)	9.82 ± 21.30	7.57 ± 19.32	13.15 ± 19.80	0.107
Hemoglobin (g/L)	79.94 ± 22.60	81.37 ± 25.25	77.83 ± 18.01	0.377
Platelet (10^9^/L)	136.55 ± 130.37	171.91 ± 138.25	84.17 ± 97.35	**<0.001**
Hematocrit	25.43 ± 5.50	26.55 ± 5.57	23.78 ± 4.99	**0.004**
Alanine transaminase (U/L)	39.79 ± 61.41	35.09 ± 34.22	46.48 ± 86.57	0.298
Aspartate aminotransferase (U/L)	45.89 ± 56.10	40.09 ± 40.92	54.17 ± 72.12	0.158
Total bilirubin (μmol/L)	68.90 ± 88.42	51.36 ± 74.43	94.88 ± 101.04	**0.008**
Direct bilirubin (μmol/L)	48.68 ± 65.67	34.60 ± 53.73	69.53 ± 76.02	**0.004**
Albumin (g/L)	28.62 ± 5.11	29.69 ± 4.91	27.02 ± 5.03	**0.003**
Creatinine (μmol/L)	105.16 ± 87.79	93.40 ± 69.31	122.57 ± 107.99	0.083
**Adverse events**				
Renal damage	18 (13.4)	9 (11.2)	9 (16.7)	0.367
Liver damage	15 (11.2)	9 (11.2)	6 (11.1)	0.980

*The bold values means p < 0.05, with a statistical significance*.

To identify the potential risk factors for 28-day mortality of CRKP BSI, we conducted univariate analyses between the 28 day-surviving and 28 day-deceased groups. The potential risk factors included the age-adjusted Charlson comorbidity index (*p* = 0.047), antibiotic exposure in the past 2 weeks [odds ratio (OR) 3.394, *p* = 0.001], APACHE II (*p* < 0.001) and SOFA scores (*p* < 0.001) on the 1st day, hospitalized days before BSI onset (*p* = 0.041), AIT (OR 0.145; *p* = 0.008), and many factors on the 4th day after BSI onset, such as APACHE II scores (*p* < 0.001), SOFA scores (*p* < 0.001), WBC counts (*p* = 0.007), the NLR (*p* < 0.001), CRP levels (*p* < 0.001), platelet counts (*p* < 0.001), haematocrit values (*p* = 0.004), total bilirubin levels (*p* = 0.008), direct bilirubin levels (*p* = 0.004), and albumin levels (*p* = 0.003). After considering the univariate relationship with outcome and clinical relevance, we then conducted a multivariate analysis of these 134 patients and found that the NLR on the 4th day (OR 1.148, 95% CI 1.076–1.225, *p* < 0.001) and antibiotic exposure (OR 3.847, 95% CI 1.322–11.196, *p* = 0.013) were significant risk factors for 28-day mortality of patients with CRKP BSI, while AIT (OR 0.073, 95% CI 0.017–0.307, *p* < 0.001) was the only independent protective factor (Table 2).

**Table 2:** Multivariate logistic regression analysis of risk factors for 28-day mortality of patients with CRKP BSI.

	**Exp (B)**	**95%CI Exp (B)**	***P*-value**
Appropriate initial therapy	0.073	(0.017, 0.307)	**<0.001**
Antibiotic exposure	3.847	(1.322, 11.196)	**0.013**
NLR on 4th day	1.148	(1.076, 1.225)	**<0.001**
APACHE II score on 4th day	1.096	(0.987, 1.218)	0.086
SOFA on score 4th day	1.020	(0.814, 1.277)	0.863

*The bold values means p < 0.05, with a statistical significance*.

**Figure 2:**
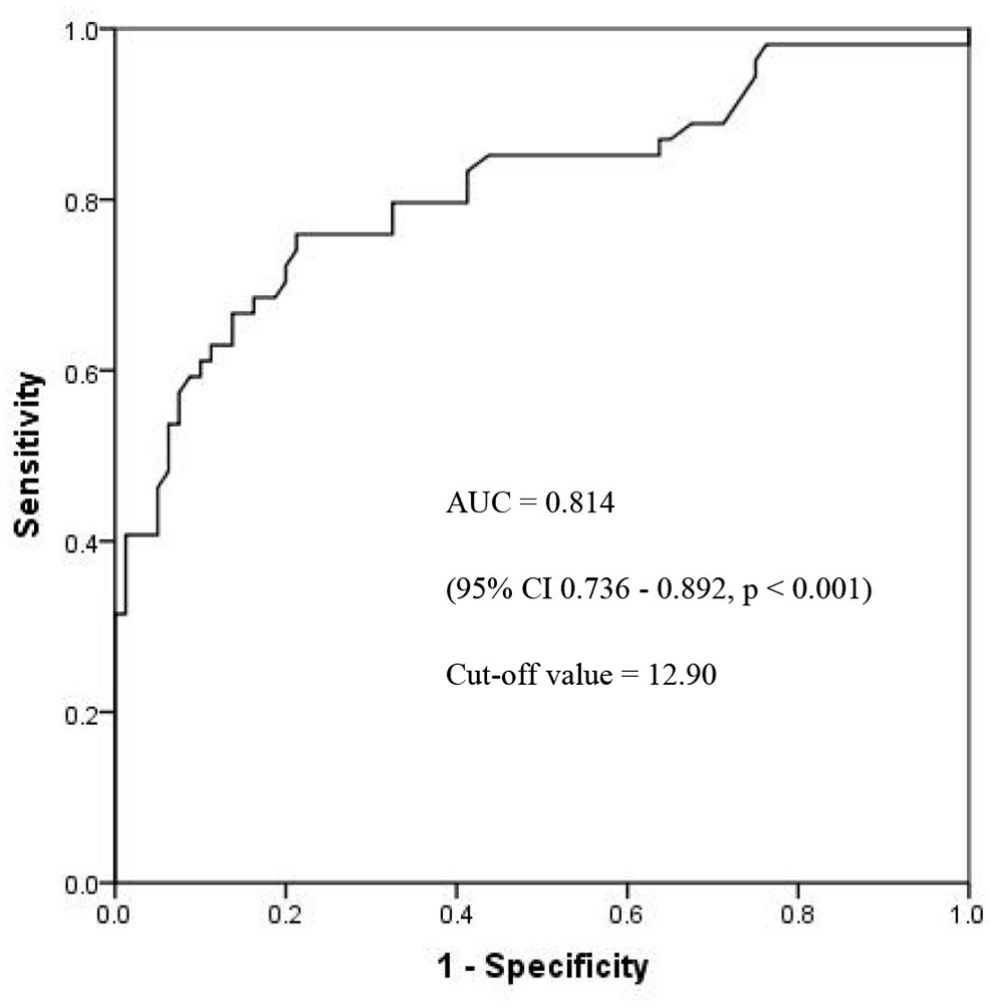
The ROC curves of NLR and 28-day mortality. ROC, receiver operating characteristic; NLR, neutrophil- to-lymphocyte count ratio; AUC, area under curves.

**Figure 3:**
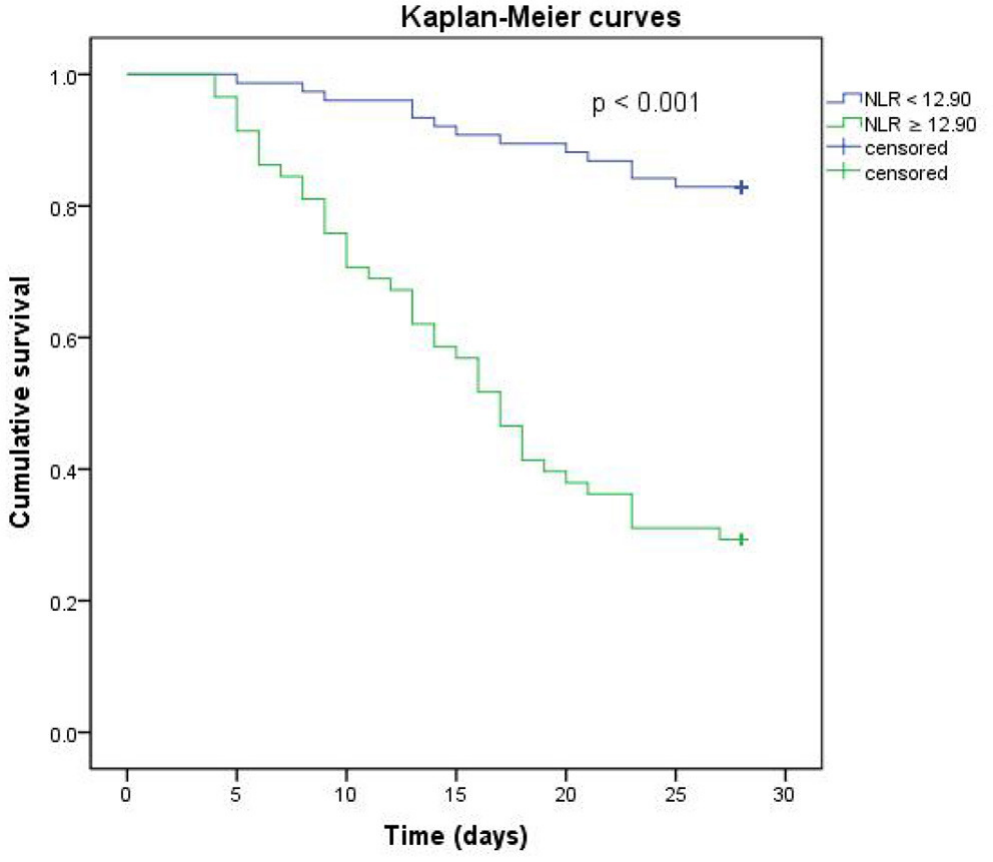
Kaplan-Meier analysis of NLR on the 4th day. The blue line represents NLR < 12.90. The green line represents NLR ≥ 12.90.

According to the results of the multivariate analysis of surviving and deceased groups, the NLR on the 4th day after onset was one of the significant risk factors for 28-day mortality (OR 1.148, 95% CI 1.076–1.225, *p* < 0.001). The receiver operating characteristic (ROC) curves of the NLR and 28-day mortality are shown in Figure 2, and the area under the curve (AUC) was 0.814 (95% CI 0.736–0.892, *p* < 0.001). In this study cohort, we found that the NLR value with the highest Youden index was 12.90, which was considered as the cut-off value of the NLR (Figure 2). All patients were divided into two groups based on their NLR on the 4th day using the 12.90 as cut-off value, and the Kaplan–Meier curves are shown in Figure 3.

### The Exposure of Antibiotics in the Past 2 Weeks Could Increase 28-Day Mortality

Together with the NLR on the 4th day, antibiotic exposure was another risk factor (OR 3.847, 95% CI 1.322–11.196, *p* = 0.013). Patients in the 28 day-deceased group had a higher rate of antibiotic exposure than those in the 28 day-surviving group (59.2 vs. 30.0%). In the 28 day-deceased group, 32 patients had a history of antibiotic use, including carbapenems (18/32), β-lactam/lactamase combinations (12/32), and tigecycline (2/32), during the last 2 weeks. In the 28 day-surviving group, 24 patients were treated with antibiotics, including carbapenems (11/24), β-lactam/lactamase combinations (11/24), quinolones (1/24), and tigecycline (1/24), before BSI onset. There were no statistically significant differences in the usage ratios between the two groups.

### Appropriate Initial Therapy Could Reduce 28-Day Mortality

According to the results of the multivariate logistic regression analysis, AIT was the only independent protective factor with an OR value of 0.073 (95% CI 0.017–0.307, *p* < 0.001). AIT included major antibiotics, such as ceftazidime/avibactam (CAZ-AVI), colistin, and tigecycline, which were active against the isolate in each case. In this study cohort, 116 of 134 (86.6%) patients received AIT, and this proportion in the 28 day-surviving group was higher than that in the 28 day-deceased group (93.8 vs. 63.5%, *p* = 0.001).

### CAZ-AVI May Offer an Important Advancement in Treating CRKP BSI With a Low NLR on the 4th Day

**Figure 4:**
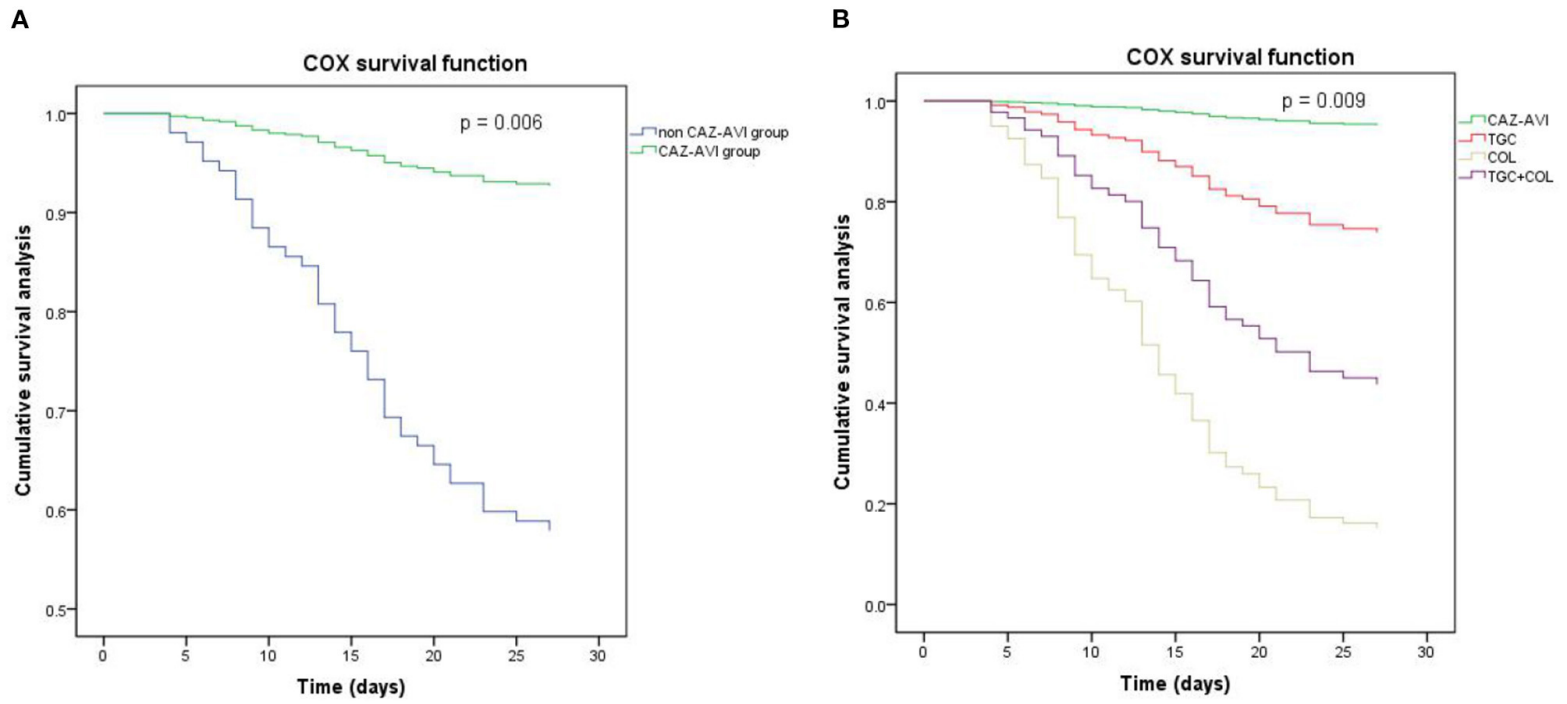
The Cox proportional hazards regression survival analysis. **(A)** CAZ-AVI vs. non CAZ-AVI (*p* = 0.006). **(B)** CAZ-AVI vs. TGC (*p* = 0.093), CAZ-AVI vs. COL (*p* = 0.003), CAZ-AVI vs. TGC + COL (*p* = 0.012). CAZ-AVI, therapy contained ceftazidime/avibactam; TGC, therapy contained tigecycline; COL, therapy contained colistin, TGC + COL, tigecycline and colistin combined therapy.

Since it has been proven that AIT is an independent protective factor for 28-day mortality of patients with CRKP BSI, we performed further analyses by dividing the patients treated with AIT into four groups: the CAZ-AVI group (14 patients), TGC group (59 patients), COL group (11 patients), and TGC + COL group (32 patients). The baseline characteristics and clinical outcomes are shown in Table 3. There were no differences in age, sex, body mass index, and underlying disease between the four groups. There was no difference in the NLR on the first day of disease in the different groups; however, there was a significant difference in the NLR on the 4th day. The Cox proportional hazards regression analysis revealed a significant difference (*p* = 0.006) between the CAZ-AVI group and non CAZ-AVI groups (Figure 4A). After dividing the non CAZ-AVI groups into the TGC, COL, and TGC + COL groups, there were no differences between the CAZ-AVI group and the TGC group (*p* = 0.093). Meanwhile, patients in the CAZ-AVI group had a lower mortality than the patients in the COL (*p* = 0.002) and TGC + COL (*p* = 0.012) groups (Figure 4B).

**Table 3:** Baseline characteristics and NLR of 116 patients treated with AIT.

	**CAZ-AVI *N* = 14**	**TGC *N* = 59**	**COL *N* = 11**	**COL + TGC *N* = 32**	***P*-value**
Age	61 (47, 65)	63 (49, 69)	69 (61, 80)	68 (61, 70)	0.125
Male	9 (64.3)	44 (74.6)	8 (72.7)	19 (59.4)	0.483
Body mass index (<18.5 or >24)	6 (42.8)	34 (57.6)	5 (45.5)	13 (40.6)	0.434
Hypertension	7 (50.0)	24 (40.7)	5 (45.5)	12 (37.5)	0.870
Diabetes mellitus	2 (14.3)	17 (28.8)	2 (18.2)	7 (21.9)	0.726
CCD	4 (28.6)	19 (32.2)	3 (27.3)	6 (18.8)	0.632
Chronic lung diseases	0	11 (18.6)	2 (18.2)	4 (12.5)	0.314
Liver failure	3 (21.4)	6 (10.2)	3 (27.3)	2 (6.2)	0.130
Renal failure	2 (14.3)	6 (10.2)	2 (18.2)	1 (3.1)	0.281
Solid tumor	4 (28.6)	23 (39.0)	4 (36.4)	11 (34.4)	0.675
Hematological malignancy	0	4 (6.8)	0	4 (12.5)	0.542
Immunosuppressive therapy	4 (28.6)	5 (8.5)	0	3 (9.4)	0.123
aCCI	3.71 ± 1.98	4.63 ± 2.31	6.09 ± 2.35	4.78 ± 2.50	0.103
NLR on the 1st day	31.48 ± 30.45	33.10 ± 41.44	25.70 ± 19.50	31.84 ± 46.35	0.958
NLR on the 4th day	9.21 ± 4.72	16.50 ± 16.66	34.60 ± 40.30	23.17 ± 31.98	**0.047**

*CAZ-AVI, ceftazidime-avibactam; TGC, tigecycline; COL, colistin; CCD, Cardiovascular and Cerebrovascular diseases; aCCI, Age-adjusted Charlson comorbidity index. The bold values means p < 0.05, with a statistical significance*.

## Discussion

The emergence of carbapenem resistance of *K. pneumoniae* is becoming challenging to treat and significantly impacts patient mortality, especially BSI (6, 7). It has been reported that drug resistance is associated with increased mortality, as patients tend to receive inappropriate empirical antibiotic therapy (9). Only few antibiotics, such as CAZ-AVI, colistin (COL), and tigecycline (TGC), have been confirmed to be effective in treating CRKP BSI *in vitro* and *in vivo*. Together with active antibiotics, early and accurate evaluation is an important method for improving outcomes. In clinical practice and previous studies, we observed that 2–3 days after initiation of the initial therapy is a recommended time point to evaluate the efficacy of the antibiotic therapy and do some adjustments if necessary in patients with BSI (15, 16). During the early period of infection, the inflammatory response can stimulate the production of neutrophils and speed up the apoptosis of lymphocytes, leading to multiorgan dysfunction (17, 18). Exploring the therapeutic effect and prognostic value of potential biomarkers in this period is important. Considering that the average time for patients to receive the first dose of appropriate initial therapy was 1.21 ± 1.32 day (116 samples) after the blood was sampled (containing the time for pathogen culture) in our center, the 3rd or 4th day after BSI onset seemed to be a target time point. A stable concentration is an important factor for antibiotics to have a therapeutic effect. Finally, we decided to use the 4th day after BSI onset as a target observation time point, as well as the 1st day of BSI onset, to ensure that we enrolled patients with stable concentrations. A suitable biomarker must provide additional information to what is presently available; it should be able to predict outcomes or evaluate the efficacy of treatment, and it should be immediately available and cost-effective (19). The present study aimed to evaluate factors, including clinical and demographic characteristics, blood biomarkers, and different therapy strategies. It was found that the NLR on the 4th day and antibiotic exposure within the past 2 weeks were independent risk factors for 28-day mortality of patients with CRKP BSI, while appropriate initial therapy was an independent protective factor.

The early phase of infection is considered a proinflammatory state mediated by neutrophils, macrophages, and monocytes with the release of inflammatory cytokines, such as tumor necrosis factor-α and interleukin 1 and 6. Neutrophils may function as killers as part of the innate response in this state with the suppression of apoptosis, causing injury (17). At the same time, lymphocyte apoptosis is increased in the thymus and spleen, leading to immune system suppression, multiorgan dysfunction, and death (18). The NLR has been used as a guide to the prognosis in various clinical conditions, such as cancer (11), ischaemic heart disease (10), and community-acquired pneumonia (20). The NLR has been observed in some studies to be more efficient than regular inflammation biomarkers in adults (14). It has also been reported that the NLR could function as a predictor of pediatric sepsis (21). Lowsby et al. evaluated the performance of the NLR as an early indicator of BSI and concluded that it may offer some diagnostic utility when taken into account as part of the overall assessment but fail to guide the clinical management of patients with suspected BSI itself (22). We conducted this study to evaluate the NLR as a predictor of the prognosis of patients infected with CRKP BSI, along with other biomarkers and therapy strategies. We found that the NLR on the 4th day after BSI onset could be an independent risk factor for 28-day mortality, and the AUC of the ROC was 0.814 with a cut-off value of 12.90. The survival rate of patients with an NLR value of <12.90 on the 4th day was significantly higher (Figure 3), which may offer a fresh insight into the evaluation of prognosis. To the best of our knowledge, this is the first study to explore the value of the NLR in predicting the 28-day mortality of patients with CRKP BSI.

According to the results of the multivariate analysis, antibiotic exposure within the past 2 weeks was an independent risk factor. The patients in the 28 day-deceased group had a higher rate of antibiotic use than those in the 28 day-surviving group. Carbapenems and β-lactam/lactamase combinations were the most commonly used. Previous studies have demonstrated that a history of antibiotic use increases the risk of CRKP infection (23), and a meta-analysis determined the OR results of previous antibiotic use (OR = 3.31), exposure to carbapenems (OR = 4.01), aminoglycosides (OR = 2.05), glycopeptides (OR = 2.40), quinolones (OR = 2.28), and anti-pseudomonal penicillins (OR = 2.67) (24). In our study, the OR of antibiotic exposure within the previous 2 weeks was 3.847 (*p* = 0.013) for 28-day mortality, while there was no difference in carbapenems, β-lactam/lactamase combinations, tigecycline, and quinolone usage between the two groups. The data showed that antibiotic exposure not only increased the risk of CRKP BSI but also increased mortality. Thus, a rational use of antibiotics can contribute to the decrease in morbidity and mortality associated with CRKP BSI.

Kohler et al. performed a meta-analysis (7 studies, 658 patients) of the relationship between AIT and mortality and concluded that AIT was a protective factor (unadjusted OR = 0.5) in both CSKP and CRKP bacteraemia (9). Another study that focused on CRKP BSI in high-risk hematological patients showed that AIT was the only independent factor able to protect against death (*p* = 0.02) (25). In the present study, we demonstrated that AIT is the only independent protective factor for 28-day mortality of patients with CRKP BSI, indicating that the use of at least one active antibiotic within 72 h can improve the prognosis. In addition to antimicrobial susceptibility tests, healthy conditions, underlying diseases, and economic burden should be considered when developing an appropriate therapy strategy, and an early evaluation of curative effectiveness is also essential. Thus, we performed further analyses of the data of patients who received AIT using a Cox proportional hazards regression model; we found that patients in the CAZ-AVI group had lower 28-day mortality rates than the non CAZ-AVI group. Furthermore, we divided the non CAZ-AVI group into the TGC, COL, and TGC + COL groups, based on the major antibiotics in the therapy strategies. Variance in therapy contributes to different outcomes. Patients who received CAZ-AVI as AITs had significantly improved 28-day mortality, compared to those with COL or TGC + COL, but no significant improvement when compared to the TGC group. We also found that there was no difference in NLR levels on the first day of CRKP BSI between the four groups; however, on the 4th day, the patients in the different therapy groups had significantly different NLRs. It was mentioned that the killer role of neutrophils and the apoptosis of lymphocytes at the early stage of infection can cause injury (17, 18), and these physiological processes can lead to changes in the NLR in the peripheral blood. Combined with clinical manifestations, the NLR may be an important factor for making decisions of appropriate therapy strategies, which can reduce the damage caused by the dysfunction of neutrophils and lymphocytes in each patient. This advantage can be observed on the 4th day of CRKP BSI. In addition to predicting the prognosis, the 4th day NLR may have the potential to evaluate the efficacy of the antibiotics at an early time. In clinical practice, physicians are required to evaluate and adjust the therapy strategy at the early stage of CRKP BSI due to poor outcomes; together with experience, a quantified marker is therefore desperately needed. From our perspective, the NLR, an available and economic marker, may have the function of indicating whether anti-infection therapy is suitable for patients, and is worthy of further study.

This study was limited by its retrospective, single-center design and small patient population. Due to the study size, the further analyses on therapy strategies might be limited. Among these 134 patients, only 3 patients were diagnosed as catheter-related blood stream infections, we failed to perform subgroup analyses for this special kind of infection. Limited by the retrospective design of our study and the missing data during the development of BSIs, we could not perform a dynamic profile analysis of NLR, which may offer more useful information.Culture-negative CRKP BSI was not considered in the present study because of prior antibiotic use, inadequate sampling techniques, or organisms that are difficult to identify. The primary endpoint we used was all-cause mortality, which may increase the influence on mortality caused by CRKP BSI.

To conclude, our study revealed that the NLR on the 4th day and antibiotic exposure within the previous 2 weeks were independent risk factors for the 28-day mortality of patients with CRKP BSI, while appropriate initial therapy was an independent protective factor. In this study cohort, we also found the NLR on the 4th day may have the potential to evaluate the efficacy of the antibiotics at an early stage and allow screening for suitable target therapy for every patient.

## Data Availability Statement

The original contributions presented in the study are included in the article/supplementary material, further inquiries can be directed to the corresponding author/s.

## Ethics Statement

The studies involving human participants were reviewed and approved by the Ethical Research Committee of Sir Run Run Shaw Hospital, College of Medicine, Zhejiang University. The patients/participants provided their written informed consent to participate in this study.

## Author Contributions

HW and YM contributed to both in the conception and design of the study and performed the statistical analysis. HW and XD organized the data together. FZ performed the strain identification and the antimicrobial susceptibility tests. HW wrote the first draft of the manuscript. YM, XD, and FZ wrote sections of manuscript. YY and YJ helped perform the analysis with constructive discussions. All authors contributed to manuscript revision, read, and approved the submitted version.

## Funding

This work was supported by the National Natural Science Foundation of China (Grant No. 81830069), the China International Medical Foundation (Grant No. Z-2018-35-2003), and the Key Research Program of the Science Technology Department of Zhejiang Province (Grant No. 2015C03046).

## Conflict of Interest

The authors declare that the research was conducted in the absence of any commercial or financial relationships that could be construed as a potential conflict of interest.

## Publisher's Note

All claims expressed in this article are solely those of the authors and do not necessarily represent those of their affiliated organizations, or those of the publisher, the editors and the reviewers. Any product that may be evaluated in this article, or claim that may be made by its manufacturer, is not guaranteed or endorsed by the publisher.

## Abbreviations

NLR: neutrophil to lymphocyte count ratio
CRKP: carbapenem-resistant Klebsiella pneumoniae
CSKP: carbapenem- susceptible Klebsiella pneumoniae
BSI: blood stream infection
OR: odd ratio
WBC: white blood cell count
CRP: C-reaction protein
ICU: intensive care unit
IQR: inter quartile range
APACHE II: Acute Physiology and Chronic Health Evaluation II
SOFA: Sepsis-related Organ Failure Assessment scores
ROC: receiver operating characteristic
AUC: the area under curve
CAZ-AVI: ceftazidime/avibactam
TGC: tigecycline
COL: colistin.
